# Is it still worth Publishing Case Reports? They are Part of our Lives

**DOI:** 10.21470/1678-9741-2020-0131

**Published:** 2020

**Authors:** Carlos A. Mestres

**Affiliations:** 1 Department of Cardiac Surgery, University Hospital Zürich, Zürich, Switzerland.; 2 Department of Cardiothoracic Surgery, The University of the Free State, Bloemfontein, South Africa.

**Respected Editor,**

I read with attention your latest editorial recently published in the Brazilian Journal of Cardiovascular Surgery (BJCVS) regarding the publication of case reports^[1]^. This is a timely, accurate and concise editorial, addressing a topic of interest. Yes, it is true that case reports (CR) have been banned from many publications in recent years due to the reasons you have clearly alluded to. I have to support what it is in this editorial. As we need metrics, you produced actual data taken from the three most internationally important journals in our field and it is clear to the readership that CR still represents a proportion of what these journals, like the BJCVS, have also published. We have also seen this in many other publications. Some reasons may be the same; in some cases, journals may need more CR load. There may always be a reason for a CR with educational value.

Other journals have designed a different strategy. The good example is that of the Journal of the American College of Cardiology (JACC). The JACC, like other journals, have evolved in a way that a family of journals has been created. As per the JACC, the Journal of the American College of Cardiology-Case Reports has been launched in June 2019^[2]^. The JACC-CR focuses only on CR. The journal name is self-explanatory. After a peer-review process, accepted cases are published under the formula which contemplates publication fees. Moreover, this tendency goes on around the world.

Therefore, once again, I have to support the essence of the wise comments delivered in this recent editorial. The statement “... Case reports and case series may be the ‘lowest’ or the ‘weakest’ level of evidence, but they often remain the ‘first line of evidence’ and is where everything begins…” holds, to me, true. What we need is a very strict peer-review process that ensures that high quality CR ultimately reach every journal’s readership.

## References

[1] Evora PRB, Braile DM (2020). Reasons to keep “case reports” alive. Braz J Cardiovasc Surg.

[2] JACC (c2020). Case Report.

